# The policy landscape and challenges of disaster risk financing: navigating risk and uncertainty

**DOI:** 10.1111/disa.12560

**Published:** 2023-01-25

**Authors:** Olivia G. Taylor

**Affiliations:** ^1^ Lecturer, Department of Geography University of Sussex United Kingdom

**Keywords:** anticipation, anticipatory action, disaster risk finance, decision‐making, risk, uncertainty

## Abstract

A more anticipatory, pre‐agreed response is a shared goal of many in the disaster management and humanitarian communities. This paper considers the emerging policy landscape of disaster risk financing (DRF), which is taken here to include mechanisms that allow agencies to act in advance of disasters occurring, as well as those that aim to respond earlier to disasters which have already happened. What they both have in common is no longer waiting for needs to become apparent before responding; however, this creates a challenge for practitioners because of the potential for acting erroneously. This paper provides a more cohesive way of understanding approaches in this policy area through the shared challenge of decision‐making under the condition of uncertainty. Drawing on expert interviews and science and technology studies theory, it sets out some recommendations on how practitioners can navigate risk and uncertainty better within DRF and in a more nuanced way.

## نبذة مختصرة

تعد الاستجابة الاستباقية والمتفق عليها مسبقًا للكوارث هدفًا مشتركًا للكثيرين في إدارة الكوارث والمجتمعات الإنسانية. تتناول هذه الورقة المشهد السياسي الناشئ لتمويل مخاطر الكوارث، والذي أعرِّفه هنا على أنه يشمل الآليات التي تسمح للوكالات بالتصرف مسبقًا عند وقوع الكوارث، وكذلك تلك التي تهدف إلى الاستجابة في وقت مبكر للكوارث التي حدثت بالفعل. القاسم المشترك بين ما سبق هو أنه لم يعد هناك حاجة لانتظار حدوث الكارثة بشكل واضح حتى تتم الاستجابة، ومع ذلك فإن هذا يخلق تحديًا للممارسين بسبب احتمالية التصرف بشكل خاطئ. تستكشف الورقة المشهد السياسي حول إطار التنمية الاقتصادية وتوفر طريقة أكثر تماسكًا، لفهم النهج في مجال السياسة هذا من خلال التحدي المشترك المتمثل في صنع القرار في ظل عدم اليقين. بالاعتماد على مقابلات الخبراء ونظرية دراسات العلوم والتكنولوجيا، تقدم الورقة توصيات حول كيفية يمكن للممارسين التعامل بشكل أفضل مع المخاطر وحالات عدم اليقين في التنمية الاقتصادية بطريقة أكثر دقة.

الكلمات المفتاحية: الترقب؛ عمل استباقي؛ تمويل مخاطر الكوارث؛ صناعة القرار؛ مخاطرة؛ ريبة؛

## 摘要

更具预期性和预先商定性的灾害响应是灾害管理和人道主义界中许多人的共同目标。本文考虑了灾害风险融资 (DRF) 的新兴政策格局，我在此将其定义为包括允许机构在灾害发生之前采取行动的机制，以及旨在更早应对已经发生的灾害的机制。他们的共同点是不再等待灾害需求变得明显后才做出反应，然而，这给从业者带来了挑战，因为有可能出现错误的行动。本文探讨了 DRF 有关的政策格局，并通过在不确定性下决策的共同挑战，提供了一种更具连贯性的方式来理解该政策领域中的各种方法。本文借鉴专家访谈和科技研究理论，为从业者如何以更细致入微的方式更好地驾驭 DRF 中的风险和不确定性提供了建议。

关键词：预期，预期性行动，灾害风险融资，决策，风险，不确定性

## Introduction

Historically, disaster risk reduction and preparedness have made up a small proportion of overall expenditure on disaster response (Kellett and Caravani, 2013). In recent years, however, movement towards a more anticipatory, pre‐agreed approach, which I refer to broadly in this paper as disaster risk financing (DRF), has become a key goal of many in the disaster response and humanitarian sectors.

A watershed year in this respect was 2021, which saw a series of key events and significant new commitments to fund risk financing. During the G7 (Group of Seven) meeting hosted in the United Kingdom in June 2021, the Governments of Germany and the UK respectively committed GBP 120 million and GBP 125 million of new financing (approximately USD 160 million and USD 140 million) for pre‐arranged disaster risk financing for vulnerable communities (UK Presidency of the G7, 2021). In September 2021, the United Nations Office for the Coordination of Humanitarian Affairs (UN OCHA) convened a high‐level event focused on anticipatory action, in partnership with the Governments of Germany and the UK, at which a number of countries and agencies further boosted their commitments to these approaches (United Nations and the Governments of Germany and the United Kingdom, 2021). For example, Germany announced its ambition to double its contribution to anticipatory action by 2022, while the Government of Ireland committed to directing approximately 25 per cent of its humanitarian funding straight to mechanisms that support anticipatory action (United Nations and the Governments of Germany and the United Kingdom, 2021, p. 2).

Sentiment regarding the current policy shift towards more anticipatory disaster financing was summed up by the current Under‐Secretary‐General for Humanitarian Affairs and Emergency Relief Coordinator, Martin Griffiths. Speaking at the High‐Level Humanitarian Event on Anticipatory Action on 9 September 2021, he stated that: ‘The humanitarian system must be as anticipatory as possible, and only as reactive as necessary’ (Griffiths, 2021, p. 2).

The significant increase in the momentum behind anticipatory and risk financing approaches has resulted in an emergent and rapidly evolving policy area. One noticeable characteristic is the complex terminology for particular mechanisms. The two most common phrases used are ‘anticipatory action’, which usually refers to anticipatory financing mechanisms implemented by humanitarian agencies, and ‘disaster risk financing’, which usually refers to mechanisms used by development financing institutions to provide rapid financing in the aftermath of a disaster.

While there are differences between the mechanisms in this area, they also have a great deal in common, specifically relating to the challenge of decision‐making when agencies are no longer waiting for disaster needs to become apparent before responding. This is critical, because it opens up decision‐making to interpretation and raises important questions regarding how decisions are made, as well as the possibility of making decisions in error. Underlying this is the challenge of navigating risk and uncertainty in decision‐making, which is the central focus of this paper.

For reasons that I explain further in the second section, I adopt a broad definition of mechanisms that fall within the scope of DRF. These are based on: (i) information about or measures of disaster risk; (ii) pre‐arranged finance and plans; and (iii) an instrument to enact a response. This definition is purposefully broad; it does not refer, for instance, to ‘anticipatory’ use of forecasts, but rather to information and measures of risk, whether that is a forecast, an expert advisory alert, or a proxy measurement of a hazard (such as windspeed) in order to inform and trigger a response. This allows diverse policy mechanisms ranging from index‐based insurance to forecast‐based financing (FbF)^1^ to be understood as different tools within the same policy landscape.

This paper explores the policy landscape of DRF and provides a more cohesive way of thinking about the different mechanisms in use, spanning those that permit agencies to act in advance of disasters occurring, as well as those that aim to respond earlier to disasters that have already happened. I unpack the central policy narratives supporting DRF, relating to efficiency and effectiveness. These demonstrate the tensions across the sector and differences in how agencies approach and define the central arguments. Next, I discuss some of the challenges to implementing DRF in practice. As a result of acting based on information which is inherently incomplete, acting in the face of uncertainty is a critical challenge confronting practitioners in this field. I bring to bear literature from science and technology studies (STS) to outline some of the ways in which both risk and uncertainty could be better understood and outline some recommendations for practitioners in this field.

### Methodology

This paper draws on qualitative research, including 27 expert policy interviews, combined with participant observation during key events in the DRF community and desk‐based reviews of policy literature. Interviews were semi‐structured in nature and organised through a mixture of purposive and snowball sampling to ensure that key agencies were represented. It is important to note that participants are categorised in a broad way as: humanitarian practitioner; donor; DRF specialist; catastrophe modeller; and researcher. This is necessary for two reasons. First, DRF is a small sector, so broad categories are important to maintain participant anonymity and to ensure that interviews are non‐identifiable. Second, this sector is composed of new collaborations of expertise, spanning policy, climate science, finance, and humanitarian practice. Table 1 lists the organisations represented in empirical data by broad descriptive category and exemplar job titles from among the categories of interview participants.

**Table 1:** Examples of organisations and job titles of research participants included in the sample

Interview number	Category	Organisations included in the category	Job title examples within the category
1	Humanitarian practitioner	Food and Agriculture Organization of the United Nations (FAO)	Crisis Anticipation Adviser
2	Humanitarian practitioner	International Federation of Red Cross and Red Crescent Societies (IFRC)	Global Coordinator FbF
3	Humanitarian practitioner		Senior Officer
5	Humanitarian practitioner	Red Cross Red Crescent Climate Centre	
9	Humanitarian practitioner	START Network	
11	Humanitarian practitioner	World Food Programme (WFP)	
17	Humanitarian practitioner		
21	Humanitarian practitioner		
23	Humanitarian practitioner		
26	Humanitarian practitioner		
10	Catastrophe modeller	Private consultants	Financial Sector Specialist
12	Catastrophe modeller	Oasis Loss Modelling Framework	
13	Catastrophe modeller	World Bank	
20	Catastrophe modeller		
4	Donor	Foreign, Commonwealth and Development Office (FCDO)	Humanitarian Affairs Officer
6	Donor	German Federal Foreign Office	Senior Desk Officer
16	Donor	United Nations Office for the Coordination of Humanitarian Affairs (UN OCHA)	
18	Donor		
7	DRF expert	Centre for Disaster Protection	Consultant
8	DRF expert	START Network	Technical Lead on Crisis Anticipation and Risk Financing
14	DRF expert	World Bank	
15	DRF expert		
22	DRF expert		
19	Researcher	German Red Cross	Adviser for Policy and Advocacy
24	Researcher	IFRC	Manager
25	Researcher	University of Reading	
27	Researcher		

**Note**: the organisations and job titles are grouped in this way to ensure that individual interviews are not identifiable.

**Source**: author.

Interviews were supplemented with attendance at key events relevant to the sector between 2018 and 2021. These included:

- the Red Cross' Global Dialogue Platform on Forecast‐based Financing, and later the Global Dialogue Platform on Anticipatory Humanitarian Action,^2^ in Berlin, Germany, in September 2018 and November 2019;
- the Global Facility for Disaster Reduction and Recovery (GFDRR)'s Understanding Risk Conference in Mexico City, Mexico, in May 2018;
- the United Nations' (UN) Global Platform for Disaster Risk Reduction in Geneva, Switzerland, in May 2019; and
- the Conference of the Parties to the United Nations Framework Convention on Climate Change (UNFCCC) in Madrid, Spain, in December 2019.

I participated in two further multi‐day virtual conferences during 2020 and 2021: the Red Cross' Virtual Global Dialogue Platform for Anticipatory Humanitarian Action in December 2020 and 2021; and the Insurance Development Forum's Virtual Summit in June 2021. Attending conferences and public sessions allowed for the triangulation of key narratives, especially conferences that spanned the climate, disaster risk reduction, and humanitarian communities. It also enabled me to follow the emergence of new approaches, vocabularies, and particular mechanisms within the DRF space.

## Disaster risk financing: background and policy landscape

DRF is emerging at a time when global humanitarian needs are reaching their highest level in decades (UN OCHA, 2020), a situation exacerbated by the COVID‐19 pandemic. The total value of unmet humanitarian appeals has been increasing over the past several years, from USD 8.9 billion in 2016 to USD 13.1 billion in 2020, excluding the total value of COVID‐19 relevant appeals in 2020, of which a further USD 5.7 billion was unmet (Development Initiatives, 2021, p. 33). This is set against the backdrop of a long‐term trend of rising global humanitarian funding over the past decade, yet the percentage of humanitarian appeal requirements that are met by funding has declined from 63 per cent in 2011 to 52 per cent in 2020 (Development Initiatives, 2021, p. 33).

Even prior to the COVID‐19 pandemic, key actors were making the case that more anticipatory financing was the only way to resolve the ongoing problem of humanitarian needs outstripping financing. For instance, Mark Lowcock, who served as the UN Under‐Secretary‐General for Humanitarian Affairs and Emergency Relief Coordinator from March 2017–June 2021, argued:

*We are now seeking almost US$27 billion for 2019, for the appeals from the UN, NGOs and others that I coordinate. We have raised almost $16 billion so far. That's a record … But it leaves a large gap. It would be nice to think we can fill the gap just by raising more money. But we can't. We also have to make the money we have go further. The best way to do that is to change our current system from one that reacts, to one that anticipates* (Lowcock, 2019, pp. 1–2).

Most recently, the confluence of COVID‐19 and existing drivers of humanitarian crises, such as conflict and climate change, has served to underline calls for more anticipatory financing, as well as increasing the coherence between development and humanitarian assistance.

While these recent pressures have drawn more attention to the need to change the status quo of disaster response, there is a longer trend that has catalysed calls for altering the paradigm of disaster response. Among participants interviewed for this research, the Horn of Africa crisis of 2011–12 was seen as a key example of the perceived failure to respond to disasters in a timely way, even when credible warning information had been available (Bailey, 2012). The slow response to this event was seen as a systemic failure across the humanitarian sector. One of the recommendations to follow was that agencies should do more to act despite uncertainty, no longer ‘waiting for certainty before responding’ (Hillbruner and Moloney, 2012, p. 1). Wider lessons learned included the need to ensure that scientific information is used better in decision‐making, and that decision‐makers act on it (Humanitarian Emergency Response Review, 2011). One research participant referred to the period of time after this crisis as representing a ‘step change’ (Interview 8, DRF specialist), which led to a focus on a more anticipatory approach and arguably laid the groundwork for DRF. Indeed, some of the findings resulted in the use of triggers for action, one of the key characteristics of DRF. As one participant explained: ‘What we have seen in the history … of humanitarian actions is there's a lot of early warning systems that have absolutely no consequence because there is no obligation to take an action based on a warning. So, what we're trying to do is to force that’ (Interview 18, donor).

To deliver this, there has been a significant enlargement of the policy landscape of DRF initiatives. In 2017, the InsuResilience Global Partnership for Climate and Disaster Risk Finance and Insurance Solutions was launched by the G7 to provide climate risk insurance for 400 million people in developing countries by 2020 (InsuResilience, 2018). In the same year, the Government of the UK launched the Centre for Disaster Protection, to provide a technical advisory for DRF, although this was not fully operational until 2019. The Centre's work spans sovereign disaster financing mechanisms to working with humanitarian agencies on risk financing (DFID, 2017),^3^ which highlights some of the increasing interconnections between sovereign and humanitarian disaster financing through DRF. In 2018, the Global Risk Financing Facility was launched in partnership with the InsuResilience Global Partnership, to pilot further disaster risk financing tools, implemented by the World Bank and GFDRR (2018).

### Defining DRF: complex terminology

This policy area has rapidly diversified and is now a complex array of agencies, mechanisms, and projects often with their own methodologies and vocabularies, leading to a complicated and sometimes confusing debate around terminology. The two most common definitions used in this wider sector are ‘anticipatory action’ and ‘disaster risk financing’. In this subsection I discuss some of the history of the different terminologies and explain why the term DRF is adopted in this paper.

‘Disaster risk financing’ as a term was initially used by the World Bank and can be traced back to the programme name for a World Bank and GFDRR stream of work on sovereign insurance, market development, and partnerships with the private sector, titled the ‘Disaster Risk Financing and Insurance Programme’ (DRFIP), which was launched in 2011. Subsequently, in their influential book *Dull Disasters?*, Daniel Clarke and Stefan Dercon (2016), who had both been affiliated with the DRFIP, argued for a more rules‐based approach to financing disaster response, by combining what they defined as: ‘A coordinated plan for post‐disaster action agreed in advance; A fast, evidence‐based decision‐making process; and Financing on standby to ensure that the plan can be implemented’ (Clarke and Dercon, 2016, p. 3). In so doing, they provided one of the first overarching definitions that could be used to describe emerging mechanisms across the sector.

With regard to definitions, it is notable that many practitioners who work in this sector use the terms DRF and anticipatory action interchangeably. This was the perspective of one humanitarian research participant who argued that FbF should be seen as one tool within a broader landscape of DRF, on the condition that wider considerations of disaster risk management are recognised: ‘The current definition of DRF from the World Bank only focusses on the response element—a bunch of instruments to ensure liquidity for response. But this is changing, it's really … looking towards holistic perspectives on disaster risk management … that's what I hope DRF will become in the future, and in that principle, in that definition, I will say that FbF is a tool within DRF’ (Interview 2, humanitarian practitioner).

Many others interviewed for this research insisted that the difference was really a semantic one. For example, one participant argued: ‘if you talk to a government or you talk to people at risk, they don't give two craps about what you call it. They care about what you're trying to do for them’ (Interview 8, DRF specialist). Another contended: ‘we're all for having an open definition of it [referring to anticipation]…. I think it's good for all of us in the sector to have something loose’ (Interview 1, humanitarian practitioner).

Nevertheless, there has been an ongoing debate about terminology in the sector, and significant resources have been invested in trying to find consensus. For example, a number of agencies, including the Centre for Disaster Protection, the Red Cross Red Crescent Climate Centre, and UN OCHA, commissioned a joint ‘thesaurus’ of anticipatory action to: ‘enable reflection on the similarities and differences in the way organizations use language associated with the concept of anticipatory humanitarian action’ and to enable mutual understanding (De Wit, 2019, p. 5). In September 2021, the newly formed Anticipation Hub^4^ hosted an event titled ‘Linking Anticipatory Action to Risk Financing’, in order to assess the connections between anticipatory action and risk financing (InsuResilience Global Partnership and the Anticipation Hub, 2021). Despite concluding that the sector needed ‘to stop silo‐approaches across the disaster management and crisis response spectrum’ (InsuResilience Global Partnership and the Anticipation Hub, 2021, p. 1), the event was clearly premised on there being a clear distinction between anticipatory action and risk financing.

As noted in the introduction, the main distinctions that are usually drawn between the two approaches are:

- the temporality of the mechanism, whether it is anticipatory or ‘ex‐ante’; and
- by what type of agency it is implemented/funded, and whether that is an actor with a humanitarian mandate.

However, I argue that this is not the most useful way to think about the mechanisms in this sector. On the first point, while a ‘more anticipatory’ (or less late) response is a key policy objective for the sector, it is not a clear defining characteristic because temporal distinctions are often difficult to apply in practice to disasters. For instance, it has long been pointed out that the phases of mitigation and preparedness ‘pre disaster’, and response and recovery ‘post disaster’, are rarely as neatly defined in practice as they seem in the disaster management cycle depicted in the disaster studies literature (Neal, 1997; Contreras, 2016). Moreover, it is difficult to ascertain the onset of impacts of many slow‐onset hazards such as droughts, which feature prominently among the risks responded to through such mechanisms, which blurs the boundary between early action and early response (Wilkinson et al., 2018). Thus, while anticipatory response is a key policy objective, this research cautions against relying on ‘anticipation’ as a defining characteristic, even though the goal of a ‘less late’ response is clear and important.

In relation to the second point, part of the desire to distinguish between humanitarian and development financing in this sector is linked to understandable concerns about the potential loss of humanitarian impartiality. This sentiment was summed up by a senior policymaker at the German Federal Foreign Office, during a panel session at the 2018 Global Dialogue Platform conference. He recommended that different approaches of risk financing ‘be kept separate so that all approaches are not mixed up…. Ultimately, humanitarian financing is obligated to human needs and not political considerations’ (German Red Cross, 2018, p. 19). However, the hybridisation of mechanisms that has occurred in the years since suggests that it is now increasingly difficult to distinguish neatly between ‘humanitarian’ and ‘development’ finance. This is exemplified by the potential use of the World Bank's Crisis Response Window to fund humanitarian response through UN OCHA's Anticipatory Action Frameworks, a partnership that was developed through the first UN OCHA Anticipatory Action pilot in Somalia in 2020 (Getliffe, 2021). Other examples of ‘hybrid’ mechanisms have also emerged, such as partnerships between insurance and humanitarian response through the START Network's (2020) ARC Replica insurance policy, and the newly launched START Ready framework (START Network, 2021). Consequently, the type of funding or implementation agency is no longer a helpful way to distinguish between anticipatory action and disaster risk financing.

As such, the definition I adopt here is a broad approach to DRF entailing: (i) information or measures of disaster risk; (ii) pre‐arranged finance and plans; and (iii) a mechanism to enact a response.

## Understanding DRF policy objectives and narratives: efficiency and effectiveness

The notion of a quicker response to a disaster, anticipating rather than reacting, is strongly intuitive. As the saying goes, ‘prevention is better than cure’, a theme oft‐repeated by the research participants. Here, I discuss the main policy narratives in DRF: that it leads to both a more efficient and effective disaster response and is therefore the way to ‘square the circle’ of increasing humanitarian response costs. I explore these narratives and how they are understood across the sector to reveal some of the underlying contestation relating to the objectives of DRF, as well as practical challenges to implementation.

First, the financing gap between growing humanitarian need and available financing is frequently cited in policy and advocacy materials as the central driver of a more efficient and effective response via DRF. It was a key point in Mark Lowcock's speech, referred to earlier, that we can no longer simply raise more money to meet humanitarian needs: ‘We … have to make the money we have go further. The best way to do that is to change our current system from one that reacts, to one that anticipates’ (Lowcock, 2019, p. 2).

Certainly, DRF is an evolution from the status quo of ‘ex‐post’ disaster response, which has been likened to the passing of a ‘begging bowl’ around donors to raise funds after a disaster happens (Clarke and Dercon, 2016). This contributes to a fragmented and politicised response that is poorly matched with post‐disaster needs, which are often contingent on funding cycles in donor countries, with little relevance to needs on the ground (Talbot and Barder, 2016). Moreover, an earlier response can avert harmful coping strategies and protect livelihoods contributing to long‐term development gains (Wilkinson et al., 2018).

However, the link between disasters and humanitarian financing needs is, in practice, more complex. As Swithern (2018) has written, there are many reasons why disaster impacts do not correlate directly with the scale of humanitarian funding appeals. In addition, a recent meta review by the World Meteorological Organization (2021) concluded that while weather‐related disasters have increased over the past 50 years, they have caused more damage but fewer deaths, mostly as a result of improved forecasting and disaster risk reduction activities.

The relative importance of the rationales for a more effective and efficient response is also complex and varies between actors in the DRF sector. For example, there has been significant investment in ‘cost–benefit’ research into various aspects of resilience programming, early action, and preparedness by several bilateral donors, in particular the UK and the United States. For instance, Cabot Venton et al. (2013) were commissioned to conduct a report into The *Economics of Early Response and Disaster Resilience* for the UK's Department for International Development (DFID), and later a similar report, *Economics of Resilience to Drought: Kenya Analysis*, was commissioned by the United States Agency for International Development (Cabot Venton, 2017). And a *Return on Investment for Emergency Preparedness Study* was produced for the United Nations Children's Programme and the World Food Programme by the Boston Consulting Group and funded by DFID in 2015 (UNICEF and WFP, 2015). However, some agencies otherwise very active in the policy space are notable because of their absence in funding such reports, especially the German Federal Foreign Office, which was an early funder of the Red Cross’ FbF work (German Red Cross, 2015).

The robustness of such ‘cost–benefit’ evidence has been scrutinised more carefully as the sector has evolved. A recent policy paper that reviewed the evidence base for anticipatory action raises the point that easily reproducible and catchy numbers produced by return on investment and cost–benefit studies ‘can obscure the quality of and underlying assumptions behind these numbers’ (Weingärtner, Pforr, and Wilkinson, 2020, p. 34). Nonetheless, the findings of such reviews were cited to this author by the research participants, even if they were sceptical about them, which demonstrates how much these studies have cut through. For example, one stated: ‘I'm a bit sceptical about … the numbers like the data that says you can act … what is it five or six times you say before it's worse than a late response’ (Interview 6, donor). This interviewee was referring to the key statistic in the Cabot‐Venton et al. (2013, p. 1) review: ‘for every early response to a correctly forecast crisis, early responses could be made 2–6 times to crises that do not materialise, before the cost of a single late response is met’.

Efficiency was also a subject of debate among the study participants. Overall, humanitarian practitioners were cautious, with one asserting that ‘the interesting thing about aid money is we want to give it away’ (Interview 4, humanitarian practitioner). Another interviewee who worked at the interface between different specialisms in DRF outlined the differences in view between humanitarian practitioners and others in the sector: ‘If we talk to humanitarian actors … in my experience some of them get the bang for the buck argument…. They get it but they're like “no, that's not what we're here for, we're here to help people“. So, you have to frame it as you could help more people with the same … amount of money’ (Interview 15, DRF expert).

While the notion that acting earlier can make responses more effective makes sense in principle, it is also notable that this has been harder to evidence across different mechanisms and hazards. In particular, the usefulness of actions that can be employed in the window of opportunity between the warning of a hazard and the impacts of the ensuing disaster being felt has been questioned. A working group titled ‘Early Actions: Why do we always end up with chlorine tablets?‘ discussed this issue during the sixth Global Dialogue Platform on FbF (German Red Cross, 2018). Chlorine tablets are regularly distributed prior to a flood or cyclone hazard and are, of course, indispensable for preventing water‐borne disease. Participants in the session pointed out, however, that they are disseminated as part of agreed protocols because they are small and easy to preposition within the time available, but as a result, they are used in preference to other actions that would be more aligned with long‐term risk reduction activities (German Red Cross, 2018).

Of course, the potential effectiveness of early actions varies significantly between hazards, something that practitioners acknowledge. Commenting on this, one humanitarian practitioner noted that while timely action will reduce human suffering, ‘it doesn't mean that the disaster will be totally prevented. Of course, it will really depend on the hazard … like for drought I'm more inclined to say that we have enough lead time…. But for a cyclone … I mean Idai^5^, we could have had the most amazing FbF in place but still the houses will be totally destroyed’ (Interview 2, humanitarian practitioner).

Thus, the use of arguments pertaining to efficiency and effectiveness of DRF varies between different mechanisms and hazard contexts, and they have evolved over time. There is an underlying recognition that acting in advance is no ‘silver bullet’ for significant efficiency savings, and does not overcome the challenge of mitigating the impacts of major hazards, while the relationships between disaster events, humanitarian financing needs, and the response are complex. However, the mutually reinforcing narrative of DRF approaches being both more efficient and effective is highly intuitive and very powerful, especially in the wider context of pressures on the humanitarian financing system. As one participant put it: ‘there is a clear understanding that disaster risk financing instruments are super essential in the future. It is clear we are going to have more disasters, and the money that is located at this moment for humanitarian action is not going to be enough for the type of events that we will have in 10, 20 years’ (Interview 23, humanitarian practitioner).

## The challenges of decision‐making in DRF

Acting based on information rather than existing needs is the key to taking a more anticipatory approach in disaster response, yet it also presents significant challenges in terms of decision‐making. As De Wit (2019, p. 34) argues in her discussion of the language used in anticipatory approaches in this sector: ‘questions around temporality have moral implications for finding a common understanding of when decisions are taken and actions planned, how you justify those choices, and how they can be funded'. This challenge was also articulated by participants in this research, such as one who explained that: ‘early action is … to a certain extent open to interpretation…. This is why it's fundamental to work on coherence and common approaches because that way we govern this, we manage this uncertainty, we manage the questions around the evidence, and we render it credible’ (Interview 5, humanitarian practitioner).

In this section I look at how questions concerning decision‐making are navigated. It is understandable that the need to justify actions taken based on DRF information is a primary concern, and this is reflected throughout the defining pillars of DRF mechanisms. Each of the components of the definition of DRF, despite variations in terminology, can be understood as contributing to a robust process for decision‐makers to use to take action. For example, the aspect which requires that financing and plans be pre‐arranged is described in one policy document as creating ‘certainty about what finance will be available’ (Montier, Harris, and Ranger, 2019, p. 4), giving disaster managers and decision‐makers the confidence to act. This was further expressed during a keynote session at the 2018 Global Dialogue Platform conference, where certainty of finance was portrayed as ‘the “glue” to assure that early action is taken ahead of a disaster based on a scientific decision‐making process’ (German Red Cross, 2018, p. 19). Moreover, the third pillar of DRF, a mechanism to trigger a response, is intended to overcome any potential inertia created by uncertainty. For instance, one participant explained its purpose to this author as follows: ‘the function of triggers is not to tell you what to do, but when to act … you're changing the default from hesitating and wondering to taking action’ (Interview 18, donor).

However, the variation in how the pillars of DRF are defined reveals the lack of consensus on the specifics of what creates a robust decision‐making process. As outlined above, I adopt a definition of DRF as requiring ‘information or measures of disaster risk’. This was chosen as a broad and encompassing criterion; to choose a more specific definition might have excluded some mechanisms. However, there are significant differences in how this component of DRF is defined across the policy literature. By way of example, Clarke and Dercon (2016, p. 3) adopt a loose definition in *Dull Disasters?*, referring to: ‘A fast, evidence‐based decision making process’. Others place more emphasis on warning information that provides a quantifiable output, such as a policy document written by practitioners from the START Network, whose definition of DRF underlines ‘quantifying risks in advance’ (Montier, Harris, and Ranger, 2019).

These differentiations point to questions about what makes information sufficiently credible to use in DRF mechanisms. This is especially important when making comparisons across different types of hazards within the remit of DRF mechanisms, which range from volcanoes to cyclones and droughts, and which require insights from diverse physical sciences—not to mention other types of humanitarian crises covered by some mechanisms, such as conflicts or migration flows. There are no clear answers to these questions, and the different definitions and methodologies adopted across the sector show that in many cases, each implementing agency is finding its own way of managing them.

### From ‘acting on uncertainty’ to ‘acting based on risk’: how risk and uncertainty are understood in DRF policy narratives

An irony in DRF is that one of the original objectives of this policy shift was to overcome the inability or unwillingness of agencies to act in the face of uncertainty, which is perceived as having been a principal aspect of the failure to respond in a timely way to past emergencies. This was one of the conclusions drawn from the Horn of Africa crisis in 2011–12, which as discussed earlier, was a significant turning point in encouraging more anticipatory, pre‐agreed approaches. In an influential review of that event, Hillier and Dempsey (2012, p. 15) contend that: ‘Early response requires acting on uncertainty’. In this policy paper, the authors discuss quantifying uncertainty and adopting a risk management approach in humanitarian response decisions, but their arguments do not shy away from acknowledging uncertainty in decision‐making based on such information. They state: ‘Forecasts involve uncertainty: they are inevitably based on data which is not totally comprehensive and are tinged with judgement; the earlier the warning, the less accurate it is likely to be’ (Hillier and Dempsey, 2021, p. 15).

However, in the years since, it is important to note how the DRF policy space has taken shape, in many cases moving towards a policy language that focuses heavily on risk at the expense of uncertainty. Indeed, this is codified in part in the term disaster *risk* financing, and it is no surprise, therefore, that the idea of acting ‘based on risk’ has become one of the defining characteristics of the paradigm shift. This is even consistent in work that spotlights humanitarian mechanisms and does not use the specific terminology of DRF, such as De Wit's (2019, p. 6) *Thesaurus*, in which she summarises the paradigm shift of anticipation as ‘acting based on risk’. The notion of ‘acting based on risk’ or a ‘risk‐based’ approach is reflected widely in the policy literature, such as in policy documents of the START Network. Quoting one of its donors: ‘We are trying to shift to a risk‐based approach instead of needs based, with more preparedness and early action’ (START Network, 2019, p. 8). And this notion is also evident in the language used in policy materials of UN OCHA in the statement released after the High‐Level Humanitarian Event on Anticipatory Action in 2021: ‘The humanitarian system must shift away from a solely reactive response to crises towards an increasingly proactive, anticipatory approach – acting on risks instead of only reacting to needs’ (United Nations and the Governments of Germany and the UK, 2021, p. 2).

Despite the sense of there being a clear distinction between acting on uncertainty and acting ‘based on risk’, one of the key findings of this research is that when asked how they thought about risk and uncertainty in their work, practitioners had very diverse understandings and attitudes. A key difference among them related to whether or not they viewed quantifiable uncertainty as a form of risk, or as a valid form of uncertainty. The former view was more commonly held by economists and social scientists. For instance, one research participant who had worked at an economic research institution said that: ‘uncertainty would be to describe the fact that you didn't know what your probability risks are’ (Interview 15, DRF expert). This reflects a ‘Knightian’ definition of risk and uncertainty—named after the economist Frank Knight—whereby risk is associated with quantifiable uncertainty and uncertainty refers to anything that cannot be numerically quantified.

In contrast, modellers and physical scientists often considered ‘quantifiable uncertainty’ to be a valid and important type of uncertainty. Indeed, this is what probabilistic modelling is designed to communicate: stating and quantifying predictive uncertainty (Gneiting, 2008). For example, one participant with a technical background explained: ‘there is [sic] two levels…. One is the uncertainty you absolutely cannot quantify because mathematically you just can't do it…. So, there is that box of stuff we can't quantify. And then there is stuff we can quantify because we actually do have some data and you can use mathematical approaches to quantify uncertainty around that data’ (Interview 13, catastrophe modeller). Another case was highlighted by a participant with a forecasting background, who described a recent situation within the FbF community where two cyclone models contradicted each other. They argued that a full understanding of uncertainty requires taking into account uncertainty that lies beyond the scope of a forecast model. In their words:

*You've got the uncertainties that you can quantify, a sort of stochastic one, so you can say like a 50 per cent chance of a flood … but you know that there's the uncertainty that you can't quantify or characterise … that the ensemble is not representing … you would have an ensemble forecast of tropical cyclones and you've got an ECMWF* ^6^ *ensemble that says one thing and a Met Office* ^7^ *ensemble that says another thing, and if they were characterising uncertainty well, then the ensembles’ spread would be overlapping in both of them. But if they both say separate things, then what do you do? Because there's uncertainty that goes beyond what that ensemble is representing* (Interview 27, researcher).

Highlighting these differences is not intended to diagnose a lack of understanding per se, but rather to show the complexity of these concepts. Risk, for example, is not a singular, objective metric ‘out there’ that can be measured in a uniform way across different settings or hazards. As I explore further below, risk and uncertainty are complicated, determined by degrees and forms of knowledge, and specific to particular hazards and contexts, hence they are difficult to convey across disciplinary boundaries.

Lastly, this research also identified a sense among practitioners that language pertaining to uncertainty was sometimes unwelcome in their work on DRF, particularly among those working in government donor agencies. For instance, one participant stated that in their view, ‘risk is seen in a very clear way in government in particular … you have risk registers etc. There's a very formalised … “how you deal with risk” manual, that we all have to comply with…. But uncertainty is seen as “I don't know the answer” and that tends to paralyse people … even the word still seems to scare people. So, it's actually better to talk about managing risk, you know, uncertainty being a risk’ (Interview 15, DRF expert).

The view that uncertainty was difficult to talk about in a public sector context was supported by another research participant. From the perspective of financial services, they pointed out that: ‘the concept of dealing with uncertainty is pretty well ingrained in the finance sector in a way that I think it is not ingrained in the public sector…. [It] is very challenging for the public sector to get its head around’ (Interview 13, catastrophe modeller).

Taken together, these factors result in a policy sector that often focuses on risk at the expense of uncertainty. In some cases, this leads to eliminating uncertainty from the policy discourse. This is evident in Mark Lowcock's two important speeches delivered in 2018 and 2019 on the subject of anticipatory humanitarian financing.^8^ In the two speeches combined, the word ‘risk’ is used a total of 36 times, but the word ‘uncertainty’ is not used at all (Lowcock, 2018, 2019). In other cases, such as during the Global Dialogue Platform conference in 2018, session convenors of one side event asserted that the use of data and triggers ‘help us eliminate uncertainty about when and how to act’ (German Red Cross, 2019, p. 22).

## What do we know? What can we predict? What can we foresee?: navigating risk and uncertainty in DRF

As demonstrated, DRF uses information about potential future hazards and disasters to take action, instead of waiting for such events to happen and responding in the aftermath. As a result, acting in the face of uncertainty is a critical challenge for practitioners, as acknowledged by Hillier and Dempsey (2012, p. 15), who argue that while reducing uncertainty is important, ‘[e]arly response requires acting on uncertainty’. This sentiment was also echoed by the Head of Country Programmes at the Centre for Disaster Protection, when speaking at a public webinar.^9^ She suggested that DRF fundamentally requires reflection about: ‘What do we know? What can we predict? What can we foresee?‘. However, practitioners interviewed in this research described many difficulties in talking about uncertainty in DRF, and as noted above, there is a tendency in formal policy spaces to focus on risk at the expense of uncertainty.

In this section I draw insights from science and technology studies to make some suggestions as to how both risk and uncertainty can be better understood in relation to DRF. STS is a diverse discipline that seeks to comprehend better the role of knowledge, specifically scientific knowledge, in policy and society. STS has explored in particular how knowledge is intimately tied up with risk and uncertainty, determining the boundaries of what we know and what we do not know (Stirling, 2007, 2009, 2010), while also highlighting how knowledge shapes our perceptions of risk and uncertainty (Lash, Szerszynski, and Wynne, 1998; Wynne, 1998). Both of these points are particularly relevant to developing a better understanding of risk and uncertainty as it pertains to DRF, nuancing what is often implied as a binary distinction between risk and uncertainty, as well as of why practitioners with different disciplinary backgrounds and perspectives approach risk and uncertainty differently.

First, it is helpful to trace back common definitions of risk and uncertainty used in both academia and practice today. One of the foundational early definitions came from the economist Frank Knight, who defined risk as anything to which we can assign numerical probabilities, whereas uncertainty is anything that cannot be constrained statistically (Knight, 1921/2006). His original theorisation was tied up with his ‘theory of profit’, in which he argued that making profit required decision‐making in the face of uncertainty, because anything which could be constrained numerically could be insured against, and thus any losses could be recuperated with insurance. Knight's distinction between risk and uncertainty is reflected in later theories on risk and uncertainty, most notably the sociologist Ulrich Beck's (1992) ‘risk society’ thesis. Beck (1992) argued that the shift from an industrial society to a risk society is defined by risks becoming increasingly ‘incalculable’. According to this view, novel ‘modernity’ risks include events such as nuclear fallouts or pandemics that are not statistically predictable and cannot be constrained by risk methodologies based on calculating likelihoods, and hence cannot be insured against. Beck (1992) concluded that such non‐insurable risks define the modern era as a ‘risk society’.

Beck's ideas have proved to be a major provocation with respect to risk, uncertainty, and politics, despite numerous critiques and iterations in thinking, such as his later work on the ‘world risk society’ (Beck, 2009), which responded to critiques of Eurocentrism and acknowledges the role of governments as a backstop insurer in times of catastrophe. One of the key aspects of Beck's argument which is relevant here, however, is his use of Knight's ideas about whether or not something is numerically predictable, and thus the use of ‘insurability’ as a key distinguishing feature between risk and uncertainty.

Other work has challenged the binary distinction between what is numerically predictable and what is not, focusing on the distinction between risk and uncertainty. Empirically, it has been shown that many modern insurance techniques blur the distinctions between calculative and non‐calculative techniques because they include aspects of ‘intuition’ and non‐quantitative approaches (Bougen, 2003; O'Malley, 2003, 2004). While insurance is seen as a stereotypically ‘risk‐based’ practice in the thinking of both Knight and Beck, many insurance practices are, in fact, characterised by educated guesswork and hunches (Bougen, 2003), where ‘knowability’ is not a clear binary.

More recent analysis of the global reinsurance industry further supports this argument. For example, Jarzabkowski, Bednarek, and Spee (2015) provide an account of reinsurance as a financial market for hedging against ‘unknown unknowns’, based on collective practices that span both technical and contextual expertise.

This complexity and nuance concerning the distinction between risk and uncertainty was highlighted during this study by participants who had come to DRF from catastrophe modelling and reinsurance. For instance, one participant commented that: ‘there is definitely a sort of, I would say intuition that builds up over time, and I think has built up with people in the industry who have been using these models for 20 years or so’ (Interview 13, catastrophe modeller). However, another participant with a modelling background felt that humanitarian practitioners were less comfortable with this complexity in modelling, stating that for the humanitarian community, ‘things are often about next year or the next three years … they want a very fixed answer, often people look very much only at the single value output and say, “Oh it's right or wrong”‘ (Interview 10, catastrophe modeller).

Going beyond the specific contours of what is calculable and what is not, it is important to concentrate instead on how knowledge is tied up with how we define risk and uncertainty, while being mindful of the complex nature of knowledge itself. Stirling's (2009) work on the condition of ‘incertitude’ is useful here, which he sees as extending beyond risk and uncertainty to encompass the conditions of ignorance, uncertainty, ambiguity, and risk. He argues that the boundaries between these different conditions are distinguished by degrees of knowledge relating to two parameters: the extent of knowledge of possible outcomes; and the extent of knowledge of the likelihoods of such outcomes (Stirling, 2009). Like others have shown in terms of the ‘fuzzy’ boundary between risk and uncertainty discussed above, Stirling (2009) asserts that the difference between what is known and what is unknown is not always as clear as one might like to think, because the nature of scientific knowledge is not linear, monolithic, or additive. Instead, knowledge is diverse, and often tacit; in many cases, knowing more does not confirm previous knowledge, but rather undermines or destabilises what we thought we knew before (Stirling, 2009).

Thus, what most risk and uncertainty scholars share is the view that risk is distinguished from uncertainty by degrees of knowledge. This insight is particularly pertinent to DRF, because it brings into question the binary between risk and uncertainty that is implied in the focus on ‘acting based on risk’ as opposed to uncertainty. The information and methodologies being used to trigger more anticipatory action might produce a numerical output, but in practice this is a lot ‘fuzzier’ than it might seem, and there is still inherent uncertainty in this process.

Second, focusing on the role of knowledge in our understanding of risk and uncertainty in DRF opens up space to consider how practitioners understand risk and uncertainty, and how this is influenced by different disciplinary perspectives and epistemologies, or ways of knowing. This is a particularly important consideration in DRF because the sector requires insights from a wide range of expertise, from hydrologists and climate scientists to actuaries and humanitarian practitioners. The resulting policy landscape is highly interdisciplinary, and this has contributed to different ways of thinking about risk and uncertainty, as discussed in the section on the challenges of decision‐making in DRF.

STS theory has again made important contributions to comprehending the role of knowledge and positionality in risk and uncertainty. For example, in an account of science and policy in the UK in the wake of the Chernobyl disaster in 1986 in the then Soviet Union, Wynne (1998) explores the advice given to Cumbrian sheep farmers and the difference in approach between a farming and scientific perspective. He contrasts lay knowledge with expert knowledge to show how epistemology is crucial to our understanding of uncertainties, demonstrating that the sheep farmers’ tacit knowledge led them to be sceptical about assumptions of predictability, prediction, and control made by the scientific community (Wynne, 1998). Indeed, the fact that epistemology and cultural factors play a significant role in determining perceptions of risk has been widely demonstrated in disaster studies literature (Bankoff, 2003; Krüger et al., 2015; Binder and Baker, 2017). However, reflexive analyses of understanding of risk and uncertainty among members of the disaster studies community itself are much less common (Hewitt, 2015).

The differences identified in this research regarding how practitioners thought about risk and uncertainty in their work further supports this statement. While the different conceptions discussed earlier are all consistent with the overarching argument that the difference between risk and uncertainty is determined by degrees of knowledge, they place emphasis in multiple ways that can cause confusion in a sector such as DRF. This study does not suggest that we should try to reconcile these perspectives in a unified way of thinking about risk and uncertainty: different objectives and scopes of work are both good reasons why natural scientists and modellers think about risk and uncertainty in a different way to economists and policymakers, for example.

While there has been significant interdisciplinary exchange and learning in this sector, such as the aforementioned *A Thesaurus for Anticipatory Humanitarian Action* (De Wit, 2019), this research recommends that practitioners engage more thoroughly with questions of knowledge and explore how risk and uncertainty are perceived and understood by both different individuals and agencies. This would help to build understanding and awareness of why risk and uncertainty mean different things to different people, how this is expressed, and how it can be better managed in DRF.

## Conclusion

In analysing the emerging policy area of DRF, this paper has made three key contributions. First, it provides a more cohesive way of defining the sector, which has a number of different terminologies associated with it, most notably in relation to ‘anticipatory action’ and ‘disaster risk financing’. While considerations such as temporality are very important, mechanisms in this wider sector have a great deal in common because of the way in which they link information about disaster risk with action, to facilitate a more timely response. This presents a shared challenge around acting based on information which is inherently incomplete, and therefore acting in the face of uncertainty. Focusing on this common problem is potentially useful for cutting through some of the terminological complexity of this emerging policy space, and drawing shared lessons, most notably about how risk and uncertainty can be better understood and navigated.

Second, the paper has discussed and unpacked the policy narratives in the sector, revealing some of the underlying contestation relating to the objectives of DRF. Although the central policy narratives of a more efficient and effective response are highly intuitive and appear to be mutually reinforcing, these goals are difficult to achieve and vary between hazard contexts and agencies. These complexities point to some of the practical difficulties in implementing DRF, which should not be underestimated.

Third, the paper has spotlighted the central challenge of DRF: acting based on information rather than on existing needs. While this is key to the potential that DRF offers in terms of a better disaster response, it also raises significant obstacles in terms of decision‐making.

Despite the obvious importance of managing uncertainty and questions concerning evidence in the DRF sphere, the paper highlights the tendency in recent years to replace discussions of uncertainty—at least in formal policy documents and speeches—with a vocabulary that concentrates narrowly on ‘risk‐based’ decision‐making. This relates in part to the sentiment among some participants interviewed in this research, especially those from the donor community, that uncertainty is difficult to recognise explicitly in their work.

In practice, however, the field of risk financing and anticipation necessarily grapples with what we know, and by corollary, what we do not know. Drawing from STS theory, the paper concludes that we should think about risk and uncertainty from the standpoint of knowledge, which bounds what is known and what is not known, and recognise that this is often much less clearly distinguishable than we would like to think.

## Acknowledgements

I would like to thank the Natural Environment Research Council in the UK for funding the SHEAR Studentship Cohort programme. I am also very grateful to the anonymous peer reviewers and editors whose comments improved this paper, as well as to all of the interview participants from across the risk financing sector for taking the time to contribute to this research.

The author declared no potential conflicts of interest with respect to the research, authorship, and/or publication of this paper.

This research was funded by the NERC Science for Humanitarian Emergencies and Resilience Studentship Cohort (SHEAR SSC), grant number: NE/R007799/1, and the SHEAR ForPAc project, grant number: NE/P000673/1.

## Data availability statement

Research data are not shared.^10^

## Endnotes

1 Detailed information on the methodology for different mechanisms within DRF are beyond the scope of this paper, but for a broad typology and explanation of different mechanisms see Willitts‐King et al. (2020).

2 This event changed name during this research, to represent the shifting focus from a particular methodology of FbF towards ‘anticipatory humanitarian action’, but the content of the conferences spanned both anticipatory action and risk financing more generally.

3 It was announced in June 2020 that the UK's Department for International Development (DFID) would merge with the Foreign and Commonwealth Office (FCO) to form a new department, the Foreign, Commonwealth and Development Office (FCDO), launched in September 2020. Here I refer to and reference ‘DFID’ when the issue at‐hand predates this change, or when the document being cited was published by DFID prior to the merger. More information about the amalgamation is available at: https://publications.parliament.uk/pa/cm5801/cmselect/cmfaff/809/80902.htm (last accessed on 20 December 2022).

4 The Anticipation Hub aims to share knowledge and experiences to jointly enhance and scale up anticipatory action globally, and brings together the German Red Cross, the International Federation of Red Cross and Red Crescent Societies, and the Red Cross Red Crescent Climate Centre. For more information, see: https://www.anticipation-hub.org/ (last accessed on 13 January 2023).

5 Referring to Cyclone Idai, a major tropical cyclone that hit Mozambique, Zimbabwe, and Malawi in March 2019.

6 ECMWF refers to the European Centre for Medium‐Range Weather Forecasts, an independent intergovernmental organisation that conducts meteorological research and operational forecasting.

7 Referring to the UK's Meteorological Office, a national weather service that performs operational forecasting.

8 The first speech, in March 2018, was titled ‘A Casement Lecture: Towards a Better System for Humanitarian Financing’, part of a high‐level series of lectures at Iveagh House, organised by the Department of Foreign Affairs, Ireland. A follow‐up was delivered at the London School of Economics and Political Science in the UK in December 2019, titled ‘Anticipation saves lives: How data and innovative financing can help improve the world's response to humanitarian crises’.

9 The recorded event, titled the ‘InsuResilience Sectoral Community Workshop: Linking Anticipatory Action to Risk Financing’, took place on 20 September 2021. Timestamp: 8.56. https://www.youtube.com/watch?v=W6ZB4p4kSgo (last accessed on 20 December 2022).

10 Owing to the nature of this study, participants were assured that any data used would remain anonymous/de‐identified and would not be shared.
